# The HapMap Resource is Providing New Insights into Ourselves and its Application to Pharmacogenomics

**DOI:** 10.4137/bbi.s455

**Published:** 2008-02-01

**Authors:** Wei Zhang, Mark J. Ratain, M. Eileen Dolan

**Affiliations:** 1 Section of Hematology/Oncology, Department of Medicine; 2 Committee on Clinical Pharmacology and Pharmacogenomics; 3 Cancer Research Center, The University of Chicago, Chicago, IL 60637, U.S.A

**Keywords:** HapMap, lymphoblastoid cell lines, genotype, gene expression, population genetics

## Abstract

The exploration of quantitative variation in complex traits such as gene expression and drug response in human populations has become one of the major priorities for medical genetics. The International HapMap Project provides a key resource of genotypic data on human lymphoblastoid cell lines derived from four major world populations of European, African, Chinese and Japanese ancestry for researchers to associate with various phenotypic data to find genes affecting health, disease and response to drugs. Recent progress in dissecting genetic contribution to natural variation in gene expression within and among human populations and variation in drug response are two examples in which researchers have utilized the HapMap resource. The HapMap Project provides new insights into the human genome and has applicability to pharmacogenomics studies leading to personalized medicine.

## Introduction

Phenotypic variation provides the raw materials for evolution by natural selection. Genetic variation together with interaction with non-genetic factors is the underlying driving force of the phenotypic changes (Nei, 2007). In the past several years, genetic studies of gene expression have treated transcript abundance itself as a quantitative trait and have mapped it to human genome polymorphisms such as expression quantitative trait loci (eQTLs) (Pastinen, Ge et al. 2006). Unlike rare or Mendelian disease, common diseases such as diabetes, cancer, depression and heart disease as well as individual responses to drug treatments have been shown to be caused by different genes or their combination with environmental factors. Various studies suggest that common DNA sequence variants in genes, especially single nucleotide polymorphisms (SNPs) are responsible for many of these examples of variation in development of diseases, responses to pathogens, chemicals and drugs (Erichsen and Chanock, 2004; Bondy, 2007; Giacomini, Brett et al. 2007; Owen and McCarthy, 2007; Rabik and Dolan, 2007). The goal of the International HapMap Project (International HapMap Consortium 2003) is to develop a haplotype map of the human genome, the HapMap, to describe the common patterns of human DNA sequence variation. The HapMap is expected to be a key resource for researchers to find genes affecting health, disease and response to drugs and environmental factors.

The exploration of quantitative variation in human populations has become one of the major priorities for medical genetics. The successful identification of variants that contribute to complex traits such as gene expression and drug response is highly dependent on reliable assays and genetic maps. In this review, we briefly introduce the publicly available HapMap resource followed by a discussion of the recent progress in two areas significantly benefited from the HapMap resource: gene expression variation studies and pharmacogenomic studies. This resource is providing new insights into the human genome and cellular make-up and its impact on our understanding of the molecular basis of variable response to drugs and regulation of gene expression. We also discuss the advantages and limitations of the HapMap resource.

## Publicly Available HapMap Resource

**Figure 1:**
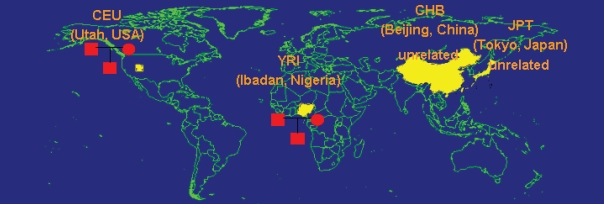
LCL samples from the International HapMap Project are derived from individuals of European, African and Asian ancestry.

Four populations were selected for inclusion in the International HapMap Project (International HapMap Consortium 2003; International HapMap Consortium 2004; International HapMap Consortium 2005; Frazer, Ballinger et al. 2007): 30 trios from Ibadan, Nigeria (YRI), 30 trios of U.S. residents of northern and western European ancestry (CEU), 45 unrelated individuals from Tokyo, Japan (JPT) and 45 unrelated Han Chinese individuals from Beijing, China (CHB) (Fig. 1). Epstein Barr virus (EBV)-transformed lymphoblastoid cell lines (LCLs) were derived from these individuals. Table 1 lists the datasets that have been made public on these populations by the HapMap Project and other laboratories. They can be grouped in three major catogories: (1) genotypic data, (2) gene expression data and (3) other phenotypic data.

### Genotypic data on the HapMap cell lines

The HapMap web site (Thorisson, Smith et al. 2005) is the primary portal to genotypic data produced as part of the International HapMap Project (Table 1). More than 4 million SNPs (with a minor allele frequency 35%) were genotyped in 270 LCLs from the four populations. Available data also include assay design information, allele frequency information, raw genotypes, and analytic results including pairwise linkage disequilibrium (LD) between SNPs and phased haplotypes. There are also public datasets for copy number variation (CNV), a structural genetic variation different from SNPs. The Database for Genomic Variants (Iafrate, Feuk et al. 2004; Redon, Ishikawa et al. 2006) contains the CNV data on 55 unrelated CEU samples using array-based comparative genomic hybridization (Albertson and Pinkel, 2003). More comprehensive CNV data on 270 HapMap cell lines have recently been made public by Stranger et al. (Stranger, Forrest et al. 2007).

### Gene expression data on the HapMap cell lines

Gene expression data using different microarray platforms have been made public through Gene Expression Omnibus (GEO) for the four populations in the HapMap Project (Table 1). Although a comprehensive comparison of these particular expression datasets has not been reported (a potential topic for researchers using these datasets), performance across microarray platforms (e.g. Affymetrix, Illumina, GE Healthcare and Agilent) has been shown to be generally reliable versus other quantitative gene expression technologies such as TaqMan assays (Canales, Luo et al. 2006). One potential problem with the use of expression microarrays is that oligonucleotide hybridization could be affected by polymorphisms located within probes (Gilad, Rifkin et al. 2005). It has been shown that sequence polymorphisms can result in many false-positives when testing for *cis* eQTLs (Alberts, Terpstra et al. 2007). A recent version of expression dataset using the Affymetrix GeneChip^®^ Human Exon 1.0ST Array took an extra step to filter out probesets affected by SNPs maintained in dbSNP (a database of SNP data curated by the National Center for Biotechnology Information) when summarizing gene expression (Zhang, Duan et al. in press).

### Other phenotypic data

Other phenotypic data such as cell growth inhibition and/or apoptosis after drug treatment and drug metabolizing enzyme activity are being generated by various laboratories that use the HapMap cell lines as experimental models (Dolan, Newbold et al. 2004; Watters, Kraja et al. 2004; Huang, Duan et al. 2007; Huang, Duan et al. 2007; Huang, Kistner et al. 2007). Published pharmacology-related phenotypic data on the HapMap samples can be accesed at the PharmGKB (Klein, Chang et al. 2001) web site (Table 1).

## Genetic Determinants of Gene Expression Variation

Gene expression as a complex trait or phenotype is believed to be a composite reflection of multiple genetic and non-genetic factors and the genetic contribution is consequently often difficult to characterize (Stranger, Forrest et al. 2005; Forton and Kwiatkowski, 2006; Zhang, Richards et al. 2007). Understanding patterns of expression variation within and among population groups will provide important insights into the molecular basis of phenotypic diversity and the interpretation of patterns of expression variation in disease. The International HapMap resource along with association studies has allowed researchers to begin to characterize the genetic contribution to gene expression variation observed between individuals and populations. Impressively, with the availability of millions of genotypes already determined for cell lines derived from major world populations, the HapMap resource has accelerated these gene expression studies.

An early study using 35 Centre d’Etude du Polymorphisme Humain (CEPH) LCLs including some HapMap samples examined ~2500 expressed genes for natural variation in gene expression and identified genes whose transcript levels differed greatly among unrelated individuals (Cheung, Conlin et al. 2003; Cheung, Jen et al. 2003). They also found evidence for familial aggregation of expression phenotype by comparing variation among unrelated individuals, among siblings within families and between monozygotic twins. These observations suggest that there is a genetic contribution to polymorphic variation in the level of gene expression. In contrast, using 60 unrelated CEU samples from the HapMap, Stranger et al. scanned 630 genes (374 expressed) for association of SNPs with inter-individual expression variation in LCLs. The signal proximal (*cis*-) to the genes of interest was found to be more abundant and more stable than those distal and (*trans-*) across statistical methodologies (Stranger, Forrest et al. 2005). Impressively, by regional association with only the HapMap SNP markers that had strong linkage evidence from a previous study (Morley, Molony et al. 2004), Cheung et al. were able to confirm the linkage results and narrow the candidate regulatory regions for many expression phenotypes of the 374 phenotypes they surveyed, while a genome-wide association using >770,000 HapMap SNPs yielded highly significant results that point to the same locations as the genome scans for about 50% of the expression phenotypes. (Cheung, Spielman et al. 2005). Recently, Bergen et al. investigated the role of *cis* sequence effects in a group of genes commonly studied in cancer research in human LCLs. Based on their results and the extensive literature, one in four genes exhibits significant *cis* sequence effects, and for these genes, about 30% of gene expression variation is accounted for by *cis* sequence variation (Bergen, Baccarelli et al. 2007).

Populations differ in prevalence of many complex genetic diseases, such as diabetes and cardiovascular disease. However, the genetic basis for population differences in clinical outcomes and risk of disease is not fully understood (Falkner, 1990; Burchard, Ziv et al. 2003; Ioannidis, Ntzani et al. 2004; Bowen, Stebbing et al. 2006; Calvo and Baselga, 2006; Huang, Duan et al. 2007). Although contributors to the differences are likely to include socioeconomic and/or environmental factors, genetic variation, including variation affecting gene expression levels, is likely to play an important role. Recently, Spielman et al. characterized variation in gene expression in cells from individuals belonging to three major population groups using the CEU, CHB and JPT samples from the HapMap Project. Their results indicate that the expression phenotype differs significantly between European-derived and Asian-derived populations for 1,097 of 4,197 genes tested. For the expression phenotypes with the strongest evidence of *cis* determinants, they found most of the variation is due to allele frequency differences at *cis*-linked regulators, suggesting that specific genetic variation among populations contributes appreciably to differences in gene expression phenotypes (Spielman, Bastone et al. 2007). Akey et al. however, suggested that batch effects could be a confounding factor when interpreting their results (Akey, Biswas et al. 2007). By characterizing patterns of natural expression variation in 16 individuals from the CEU and YRI samples, Storey et al. found extensive variation in gene expression levels and estimated that approximately 83% of genes are differentially expressed among individuals and that approximately 17% of genes are differentially expressed between the two populations. Furthermore, by decomposing total gene expression variation into within- versus among-population components, they found that most expression variation is due to variation among individuals rather than among populations, which parallels observations of extant patterns of human genetic variation (Storey, Madeoy et al. 2007). In addition to confirming that common variants account for a substantial fraction of the between-population gene expression variation, Zhang et al. were able to evaluate the contribution of genetic and non-genetic factors to the observed population differences using 176 CEU and YRI samples (Zhang, Duan et al. in press). The above studies were performed to associate expression phenotype with one form of genetic variation, namely, SNPs. In contrast, Stranger et al. determined the overall contribution of SNPs and copy number variants (CNVs), which belong to another type of common genetic variation (i.e. structural variation) to expression phenotype by performing association analyses of expression levels of 14,925 transcripts with SNPs and CNVs in individuals of the four populations from HapMap Project. They found that SNPs and CNVs captured 83.6% and 17.7% of the total detected genetic variation in gene expression, respectively, but the signals from the two types of variation had little overlap, suggesting interrogation of the genome for both types of variants may be an effective way to elucidate the variation in complex phenotypes and human diseases (Stranger, Forrest et al. 2007).

In addition to many differences between populations in transcriptional and translational regulation of genes, alternative pre-mRNA splicing (AS) is also likely to play an important role in regulating gene expression and generating variation in mRNA and protein isoforms. It has been estimated that between one-third and two-thirds of all human genes undergo AS (Sorek, Shamir et al. 2004) and the disruption of specific AS events has been implicated in several human genetic diseases (Faustino and Cooper, 2003). Using 22 unrelated CEU samples from the HapMap Project, Hull et al. demonstrated that six out of 70 exon-skipping events they identified were consistently different in splicing pattern between individuals, with a highly significant association between splice phenotype and neighboring SNPs. Their findings suggest that phenotypic variation in splicing patterns is determined by the presence of SNPs within flanking introns or exons (Hull, Campino et al. 2007). Using a comprehensive exon-targeted microarray, Kwan et al. examined individual-specific alternative splicing in 60 unrelated CEU samples from the HapMap Project as well as 14 more CEPH samples. They showed that their approach can detect both annotated and novel alternatively spliced variants, and that such variation among individuals is heritable and genetically controlled (Kwan, Benovoy et al. 2007).

## Application to Pharmacogenomics

It has been especially difficult to dissect genetic contribution to common, polygenic diseases as well as other clinical phenotypes such as drug response in which multiple genetic and environmental factors may interact with each other. It is now widely accepted that association studies offer greater statistical power over linkage in detecting genetic effects underlying these complex traits when the causative variant is common in the population (Risch and Merikangas, 1996). Besides being used in traditional candidate gene approaches, the HapMap resource and technology for genome-wide analysis have enabled an unbiased approach to detect clinically important genetic factors that determine drug effectiveness and side effects between patients (Deloukas and Bentley, 2004; Andrawiss, 2005).

Clinical drug responses reflect not only properties intrinsic to the target cell but also host metabolic properties, drug-drug interactions, and pharmaco-kinetics as well as population, gender, age and other environmental factors. To evaluate population and gender effects and to begin to understand how genetic variations contribute to these effects, Huang et al. used CEU and YRI trios from the HapMap Project to study the population- and gender-specific differences for cytotoxicity following treatment with carboplatin, cisplatin, daunorubicin, and etoposide (Huang, Kistner et al. 2007). They observed large inter-individual variance in cytotoxicity as represented by IC_50_ (the drug concentration resulting in 50% cell growth inhibition) values for the four drugs for both the YRI and CEU samples. They also reported drug response differences between the two populations for two of the drugs they tested as well as a significant difference between females and males in the YRI samples for the two platinating agents. Differences in sensitivity to drugs may be explained, to some extent, by differences in gene expression between males and females (Zhang, Bleibel et al. 2007) or differences between populations (Zhang, Duan et al. in press). Their results show that population and gender may affect risk for toxicities associated with certain chemotherapeutic agents.

One of the major goals of pharmacogenomic studies is to identify genetic factors responsible for drug effectiveness and side effects among patients. When the candidate gene is known, Jones et al. demonstrated that the HapMap resource is useful for pharmacogenetic discovery by using HapMap cell lines and HapMap SNPs to test whether a thiopurine methyltransferase (TPMT) polymorphism could be identified as predicting TPMT phenotype (Jones, Yang et al. 2007). However, challenges remain for definitive gene identification when an unsupervised genome-wide approach is employed. Recently, Huang et al. used a comprehensive microarray platform to measure gene expression levels of the CEU and YRI trios from the HapMap Project and performed genome-wide association studies with ~380,000 highly informative HapMap SNPs to successfully identify genetic factors that contribute to the cytotoxicity of cisplatin and etoposide, which are widely prescribed anticancer agents (Huang, Duan et al. 2007; Huang, Duan et al. 2007). Utilizing the HapMap resource, these authors were able to use a genome-wide approach to evaluate associations between 1) genotype and drug response, 2) genotype and gene expression, and 3) gene expression and drug response. The HapMap resource has allowed these authors to find significant SNPs associated with both baseline gene expression and relative drug response. Therefore, genotype, gene expression and sensitivity to drug information can be used to identify genetic variants responsible for drug response that act through expression. Clearly, this level of integration of genotype, expression data and other phenotypic data could not be possible without a HapMap-like resource. In addition, these results will be useful to other investigators studying these lines, as the drug response data are also publicly available. Though further functional validation experiments are necessary to confirm the findings, Huang et al. showed that utilization of the HapMap resource impressively narrowed down the number of candidate genetic variants from hundred of thousands to just a handful that explain a significant percentage of the human variation in drug sensitivity. They hypothesize that each drug has a “pharmacogenetic signature” that explains most of the variation in drug sensitivity. These pharmacogenetic signatures can be integrated into clinical studies to determine whether they predict for patients at risk for chemotherapeutic-induced toxicities or response. The goal is to improve responsiveness and reduce adverse events associated with these highly toxic drugs.

## Advantages and Limitations of the HapMap Resource

There are several advantages of the HapMap resource. Investigators can utilize extensive genotype, gene expression and other phenotypic data on the 270 HapMap samples to perform various genome-wide scans for studies that do not require *a priori* knowledge, as well as more directed studies using a candidate gene approach. The HapMap samples provide an *in vitro* model for studies of the major world populations, i.e. European, African and Asian (including Chinese and Japanese). Another advantage to using these samples in pharmacogenomic studies of drug targets is that a cellular approach avoids the complexities of *in vivo* pharmacokinetic variability. Thus, genetic variation important in variation in pharmacodynamic effects can be teased out. Furthermore, such *in vitro* studies are less expensive and require less time than similar studies in human subjects, which would also need to consider the potential risks of the drugs being tested. Chemotherapy, for example, is associated with severe toxicity and therefore cannot be given to unaffected family members for genetic studies. A number of tools and techniques are now available to increase the ease of utilizing the HapMap resource, including tools for viewing and analyzing haplotype and LD data, identification of optimal sets of haplotype tagging SNPs, drawing links between associated SNPs and putative causal alleles or simply viewing LD and haplotypes across a gene or region of interest (Barnes, 2006).

However, there are some limitations of the HapMap resource. LCLs represent just one human tissue type, therefore it may not reflect tumor response or sensitivity of target tissue of known toxicity. It has been estimated that only ~50–60% of human genes are expressed in LCLs (Cheung, Conlin et al. 2003). A more comprehensive understanding of variation of complex traits or phenotypes such as gene expression requires cell lines derived from other tissues, because for example it has already been known that many pharmacology-related genes have distinct tissue-specific expression patterns (Zhang, Liu et al. 2007). Because of the fact that the EBV-transformation of LCLs from the CEU and other HapMap samples (YRI, CHB, JPT) happened over 20 years apart (Dausset, Cann et al. 1990; International HapMap Consortium 2003), certain non-genetic factors, such as the EBV strains used for transformation or the number of freeze/thaw cycles, could lead to differences in gene expression between these samples, thus the comparative results using these samples could be confounded by non-genetic factors. An appropriate approach that takes consideration of these effects, therefore, is recommended for these studies (Akey, Biswas et al. 2007). Studies suggest EBV transformation may affect the expression of some genes and certain biological processes in LCLs (Liu, Chen et al. 2004; Tobollik, Meyer et al. 2006). Therefore, interpretation of results using these cell lines may be biased by this effect.

## Conclusion and Outlook

The HapMap resource has allowed population-based studies regarding the contribution of genetic factors to variation in complex traits or phenotypes such as gene expression, drug response and susceptibility/cause of common disease in humans, as well as other studies in population genetics and evolution. For example, recent progess using the HapMap resource has begun to illustrate the contribution of common genetic variants to natural variation in gene expression within and among major world populations. Studies like these are providing new insights into the human genome and cell biology, thus changing our view of ourselves. The HapMap resource is also facilitating pharmacogenomic studies to identify genetic variants important in gene expression and sensitivity to chemotherapy, thus lending the promise of personalized medicine in the future.

Clearly, recent studies using the HapMap resource have shown its applicability to detect common genetic variants important for variation in complex traits or phenotypes such as development of common diseases (Ait Yahya-Graison, Aubert et al. 2007; Barnes, Grant et al. 2007; Kakiuchi, Ishiwata et al. 2007; Salonen, Uimari et al. 2007) and the two areas of focus in this review: gene expression and drug response. However, the HapMap resource will become an even more powerful tool if it is integrated with other resources such as a full catalogue of functional variants, databases of genetic elements (e.g. transcript factor binding sites, exonic splicing enhancers and microsatellites) and databases of functional annotations (e.g. gene ontology and known pathways). Since the current HapMap genotypic data is comprised of ~4 million common SNPs, it remains a challenging task to identify rare or untyped SNPs using this resource. Therefore, deep resequencing or a denser genotyping microarray may be needed to uncover more SNPs in the HapMap samples. One effort to resequence the HapMap samples is the coordination of the HapMap Project and the ENCODE (ENCyclopedia Of DNA Elements) Project (ENCODE Project Consortium 2004). The latter aims to identify all functional elements in the human genome and involves resequencing of 10 500Kb HapMap-ENCODE regions in 48 unrelated individuals (16 YRI, 16 CEU, 8 CHB and 8 JPT) using a PCR-based method. Approximately 20,000 SNPs were identified in the HapMap-ENCODE regions. Some of these were already represented in dbSNP, while others were discovered during the resequencing. Genotype data were obtained from these SNPs of all 270 HapMap samples. The candidate gene approach, which requires *a priori* knowledge of certain biological processes or pathways, can be complemented by targeted deep resequencing to discover rarer variants, while the whole-genome approach using the HapMap resource can be used to identify new targets of interest in an unbiased way. Finally, as more and more genotypic and phenotypic data on the HapMap samples are made publicly available by researchers worldwide, sharing and cross-referencing these datasets will be difficult. Therefore, it is evident that appropriate bioinformatics techniques and statistical methods need to be developed so that researchers have the opportunity to comprehensively incorporate the complex networks of various relationships (e.g. gene-environment, gene-drug, gene-gender, gene-age, *cis*- or *trans*- regulators) that affect complex traits.

## Figures and Tables

**Table 1:** Publicly available genotypic, gene expression and pharmacology-related data on the HapMap cell lines.

Date type		GEO ID	Technology platform	Samples	No. Of targets	Web site	Reference
Genotype	SNPs		Affymetrix 100K and 500K SNP Arrays; Perlegen	90 CEU, 90 YRI, 45 CHB and 45 JPT	~4 million	http://www.hapmap.org/	Thorisson, Smith et al. 2005; Frazer, Ballinger et al. 2007
	SNPs	GSE5013	Affymetrix 500K EA Array	90 CEU, 90 YRI, 45 CHB and 45 JPT	~474,000	http://www.ncbi.nlm.nih.gov/geo/ http://www.hapmap.org/	Redon, Ishikawa et al. 2006
	CNV loci		Spectral Genomics 1 Mb Human BAC Array	55 CEU	~2,600	http://projects.tcag.ca/variation/	Iafrate, Feuk et al. 2004
	CNV loci		Whole Genome TilePath Array	90 CEU, 90 YRI, 45 CHB and 45 JPT	~27,000	http://www.sanger.ac.uk/humgen/cnv/data/	Stranger, Forrest et al. 2007
Expression	Transcripts	GSE5859	Affymetrix Human Focus Array	60 CEU, 41 CHB and 41 JPT	~8,500	http://www.ncbi.nlm.nih.gov/geo/	Spielman, Bastone et al. 2007
	Transcripts	GSE7792 GSE7851†	Affymetrix Human Exon Array	87 CEU and89 YRI	~18,000	http://www.ncbi.nlm.nih.gov/geo/	Huang, Duan et al. 2007; Zhang, Duan et al. in press
	Transcripts	GSE7236	Affymetrix Human Focus Array	8 CEU and8 YRI	~8,500	http://www.ncbi.nlm.nih.gov/geo/	Storey, Madeoy et al. 2007
	Transcripts	GSE6536	Illumina Sentrix Human-6 Expression BeadChip	60 CEU, 60 YRI, 45 CHB and 45 JPT	~47,000	http://www.ncbi.nlm.nih.gov/geo/	Stranger, Forrest et al. 2007; Stranger, Nica et al. 2007
Pharmacology-related Phenotype	Drug response, etc.			Various		http://www.pharmgkb.org/	Klein, Chang et al. 2001

† A version filtered out probesets affected by SNPs.
